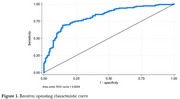# Volumetric alterations of subcortical grey matter as diagnostic indicators for Dementia

**DOI:** 10.1002/alz70856_101200

**Published:** 2025-12-25

**Authors:** Fatemeh Tabassi Mofrad, John Gallacher

**Affiliations:** ^1^ University of Oxford, Oxford, United Kingdom; ^2^ University of Oxford, Oxford, Oxfordshire, United Kingdom

## Abstract

**Background:**

Dementia, characterized by debilitating cognitive functions, has currently affected over 55 million people worldwide, highlighting the need for urgent diagnostic and therapeutic strategies; yet the neuropathological mechanisms underlying Dementia are not comprehensively addressed nor are brain structural signature associated with Dementia fully documented.

**Method:**

In order to examine volumetric alterations of subcortical grey matter as diagnostic biomarkers, we used the T1‐weighted structural brain scans data of 152 Dementia patients and 152 healthy individuals from the UK Biobank; we focused on the volumes of the Brain Stem, the Thalamus, the Caudate, the Putamen, the Amygdala, the Hippocampus, and the Pallidum and performed a multifactor MANOVA to explore differences in the combined mean of the grey matter volumes (GMVs) between participants.

**Result:**

In Dementia patients, we observed significant shrinkage of the GMVs in the Brain Stem, in the left and right Amygdala, and Hippocampus; interestingly, we also detected higher GMVs in the left and right Caudate and Pallidum, which is in fact due to neuroinflammation. To examine the diagnostic indications for Dementia based on the volumetric alterations of subcortical areas, Receiver Operating Characteristic (ROC) curve analysis was performed also by including age and biological sex factors. According to the area under the curve, the diagnostic accuracy of the model in discriminating the Dementia patients from the healthy participants is at the considerable rate of 82% (Figure 1).

**Conclusion:**

Our investigation into the GMVs of subcortical areas revealed that in Dementia patients both the atrophy and the inflammation characterize pathological alterations, uncovering the consequences of sustained neuroinflammation on brain tissue and the resulting pathogenesis of Dementia. Besides, based on the ROC curve the GMVs of subcortical areas serve as a reliable biomarker for early detection and diagnosis of Dementia in clinical settings, facilitating timely interventions.